# Development and evaluation of a PLGA/TiO_2_ nanocomposite for periodontal applications: Synthesis, characterization, and cytocompatibility with human gingival fibroblasts—An in vitro study

**DOI:** 10.34172/japid.026.3939

**Published:** 2025-12-22

**Authors:** Atabak Kashefimehr, Samira Mohammad Mirzapour, Motahare Sharifyrad, Samira Beiraghisalek

**Affiliations:** ^1^Department of Periodontics, Faculty of Dentistry, Tabriz University of Medical Sciences, Tabriz, Iran; ^2^Pharmaceutical Nanotechnology Research Center, Zanjan University of Medical Sciences, Zanjan, Iran

**Keywords:** Biocompatible materials, Gingival fibroblasts, Nanocomposites, Periodontal regeneration, Polylactic-co-glycolic acid, Titanium dioxide

## Abstract

**Background.:**

Titanium dioxide (TiO_2_) nanoparticles exhibit promising antibacterial and structural properties but raise concerns of cytotoxicity in oral soft tissues. Incorporating TiO_2_ into biocompatible polymers such as poly(lactic-co-glycolic acid) (PLGA) may reduce these adverse effects and improve clinical applicability in periodontal therapy.

**Methods.:**

TiO_2_ nanoparticles were synthesized via a modified sol–gel method and incorporated into PLGA matrices with and without polyvinylpyrrolidone (PVP) as a stabilizer. The formulations were characterized using Fourier-transform infrared (FTIR) spectroscopy, field emission scanning electron microscopy (FESEM), thermogravimetric analysis (TGA), dynamic light scattering (DLS), and x-ray diffraction (XRD). Cytocompatibility was evaluated using the MTT assay on HGF cells across concentrations of 1–100 μg/mL after 24 and 48 h.

**Results.:**

TiO_2_ nanoparticles alone induced significant, dose- and time-dependent cytotoxicity, reducing cell viability to ~45% at 100 μg/mL after 48 h (*P*<0.01). Pure PLGA maintained>90% cell viability at all tested concentrations. The PLGA/TiO_2_ nanocomposite showed markedly reduced cytotoxicity, maintaining>80% viability even at 100 μg/mL. Incorporation of PVP improved nanoparticle dispersion, as confirmed by FESEM and DLS, and enhanced the thermal stability of the composite.

**Conclusion.:**

Encapsulation of TiO_2_ within a PLGA matrix significantly mitigated cytotoxicity while preserving structural stability, with PVP further improving homogeneity and heat resistance. These findings highlight the potential of PLGA/TiO_2_ nanocomposites as safe and effective candidates for periodontal wound dressings, implant coatings, or regenerative scaffolds.

## Graphical Abstract

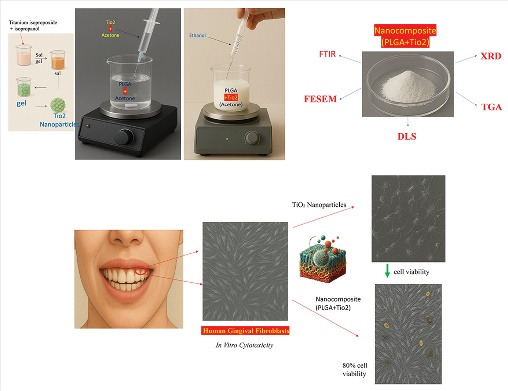

## Introduction

 Periodontal wound healing requires biomaterials that not only protect surgical sites but also actively enhance regeneration, as impaired healing increases the risk of microbial colonization and tissue failure. This dynamic biological process involves a complex sequence of cellular activities, including inflammation, matrix remodeling, and tissue formation.^1,2^ Among the key players in this process are fibroblasts, which populate the wound site within the first few days and actively contribute to collagen and elastic fiber synthesis. By replacing the provisional extracellular matrix with a more structured, collagen-rich scaffold, fibroblasts support the structural integrity and function of regenerating periodontal tissue.^3^ Additionally, they are responsible for producing a variety of cytokines and growth factors that regulate inflammation and tissue repair. Therefore, understanding the biological behavior of gingival fibroblasts is critical for developing new wound-care materials to optimize periodontal regeneration.^4^

 In clinical settings, periodontal surgical wounds are often protected with dressing materials or packs that minimize trauma during chewing and reduce postoperative bleeding. However, conventional dressings typically lack therapeutic properties and offer no active support to the healing process.^5^ Delayed or inadequate healing increases the risk of bacterial colonization, prolonged inflammation, and scar tissue formation. As such, there is a growing demand for bioactive wound dressings that not only protect but also actively promote healing.^6^

 Nanotechnology offers promising strategies to enhance wound healing. Incorporating metallic nanoparticles into wound dressings has been shown to improve antimicrobial efficacy and promote tissue regeneration.^7,8^ While nanoparticles such as silver, zinc oxide, and titanium dioxide (TiO₂) have demonstrated antibacterial properties, the efficacy of TiO₂ is generally lower and often dependent on ultraviolet (UV) light activation—unlike Ag and ZnO, which exhibit intrinsic antimicrobial activity. Nevertheless, TiO₂ remains attractive due to its chemical stability and mechanical strength, but its cytotoxicity toward soft tissue cells—particularly fibroblasts—represents a significant limitation for oral applications. This toxicity is primarily attributed to the generation of reactive oxygen species (ROS), which disrupt membranes, induce oxidative stress, and damage intracellular organelles.^9,10^

 Nanocomposites offer a strategic solution by combining nanoparticles with biocompatible polymer matrices. In this design, the nanoparticles maintain their antibacterial function while the polymer matrix limits their direct toxicity to host cells. Fibroblasts, as the most abundant cells in the periodontal ligament, are particularly important in connective tissue regeneration and must be preserved in any therapeutic system.^11^

 Poly(lactic-co-glycolic acid) (PLGA) is one of the most widely used biodegradable polymers in biomedical applications. It is approved by both the US Food and Drug Administration (FDA) and the European Medicines Agency (EMA), and has been employed in controlled drug delivery and resorbable implants since the 1980s.^12^ Due to its biocompatibility, degradation behavior, and mechanical adaptability, PLGA is an excellent candidate for composite scaffolds. To further enhance its biological performance, PLGA is often combined with synthetic or natural inorganic materials. Previous research, including our study on PLGA-ZnO nanocomposites, has demonstrated that polymer matrices can significantly reduce the cytotoxic effects of reactive metal oxide nanoparticles on gingival fibroblasts.^13,14^ TiO₂ nanoparticles have attracted attention in dental applications due to their excellent antibacterial properties, chemical stability, and mechanical strength. However, TiO₂ has also been associated with dose- and time-dependent cytotoxicity, particularly when used in free nanoparticle form, raising concerns about its safety for soft tissue applications. This cytotoxicity is primarily attributed to the generation of ROS, which can disrupt cellular membranes, induce oxidative stress, and damage intracellular organelles.^15^ Encapsulation of TiO₂ within PLGA is therefore hypothesized to mitigate cytotoxicity while preserving antibacterial activity, making it highly relevant for periodontal wound healing.

 The present study aimed to synthesize and characterize a PLGA/TiO₂ nanocomposite with or without polyvinylpyrrolidone (PVP) as a stabilizer, in which encapsulation within the PLGA matrix is designed to reduce the direct cytotoxicity of TiO₂ nanoparticles. The nanocomposite was evaluated for its physicochemical properties and in vitro cytocompatibility on human gingival fibroblasts.

## Methods

###  Materials

 PLGA (50:50, Mw ~48,000 Da), titanium (IV) isopropoxide, PVP, ethanol, acetone, hydrochloric acid (HCl), 3-(4,5-dimethylthiazol-2-yl)-2,5-diphenyltetrazolium (MTT), fetal bovine serum (FBS), dimethyl sulfoxide (DMSO), Dulbecco’s Modified Eagle Medium (DMEM), and deionized water were obtained from Sigma-Aldrich or Merck. All the chemicals were of analytical grade and used without further purification. Human gingival fibroblasts (HGFs) were acquired from a certified national cell bank.

###  Synthesis of TiO ₂ Nanoparticles

 TiO₂ nanoparticles were synthesized using a modified sol–gel method. Titanium isopropoxide was mixed with isopropanol in a 1:4 molar ratio under constant stirring on a magnetic hot plate to obtain a homogeneous solution. In a separate beaker, deionized water was mixed with isopropanol in equal volume, and the mixture was stirred magnetically for 5 minutes. The second solution was then added dropwise to the first, yielding a milky white suspension. The pH of the solution was carefully adjusted to ~4 using hydrochloric acid, added dropwise while continuously monitoring with a pH meter. The mixture was stirred for several hours and subsequently sonicated for 30 minutes to ensure complete hydrolysis and condensation. The resulting sol was allowed to age for 48 hours, during which it gradually transformed into a viscous gel. The gel was dried in an oven at 120 °C for 2–3 hours and then calcined in a furnace at 600 °C for 5 hours to obtain crystalline TiO₂ nanoparticles. The synthesized nanopowder was further characterized using field emission scanning electron microscopy (FESEM) and dynamic light scattering (DLS).^16,17^

###  Preparation of Nanocomposite Formulations

 PLGA/TiO₂ nanocomposites were prepared using PLGA 50:50, Resomer RG 504H, with a molecular weight of 48,000 g/mol (Sigma-Aldrich) as the polymeric matrix. Acetone was employed as the solvent and ethanol as the anti-solvent. Initially, 200 mg of PLGA was dissolved in 10 mL of acetone and stirred at room temperature for 15 minutes. Separately, TiO₂ nanoparticles (~0.55% w/w) were dispersed in 1 mL of acetone. The dispersed TiO₂ solution was then added dropwise to the PLGA solution under continuous stirring at 1200 rpm, followed by an additional 30 minutes of stirring. Subsequently, 15 mL of absolute ethanol was added to induce precipitation. To prevent agglomeration, the resulting PLGA/TiO₂ mixture was slowly added to an 0.05% (w/w) PVP solution prepared in 40 mL of deionized water while stirring at 1200 rpm. The final solution was left at room temperature to allow solvent evaporation, yielding the nanocomposite precipitate. The chemical structure of the nanocomposite was confirmed by Fourier-transform infrared (FTIR) spectroscopy, and its morphology was examined using FESEM.^18^

###  Characterization Techniques

- FTIR analysis: Functional groups were assessed using the KBr pellet method, recorded between 4000–400 cm ^-1^.
- FESEM: Morphological features were visualized after gold sputtering at various magnifications.
- DLS: Hydrodynamic size of TiO₂ nanoparticles
- DLS/Zeta potential: Hydrodynamic size and surface charge (zeta potential) of TiO₂ nanoparticles, PLGA/TiO₂ nanocomposites, and PVP-containing/without-PVP formulations were measured using DLS in deionized water.
- X-ray diffraction (XRD) analysis: Crystalline phases of TiO₂ and its incorporation into the PLGA matrix were confirmed by XRD, and possible changes in crystallinity or peak shifts after composite formation were evaluated.
- Thermogravimetric analysis (TGA): Thermal stability was assessed from 25 to 600°C under a nitrogen atmosphere.

###  In Vitro Cytotoxicity (MTT ASSAY)

 Cytotoxicity of the PLGA/TiO₂ nanocomposite was evaluated using human gingival fibroblasts via the MTT assay. The cells were cultured in DMEM supplemented with 10% FBS, 100 U/mL penicillin, and 100 μg/mL streptomycin at 37 °C under 5% CO₂ and 95% air. After two days, non-adherent cells and culture medium were discarded and replaced with fresh medium. Half of the medium was refreshed every three days. The cells were seeded in 96-well plates and treated with various concentrations of the polymeric nanocomposite, TiO₂ nanoparticles, and PLGA. At 24 and 48 hours, 10 μL of MTT solution (5 mg/mL) was added per well. The plates were incubated in the dark for 3–4 hours to prevent photodegradation of tetrazolium salts. Formazan crystals were dissolved with 100 μL DMSO per well, and absorbance was measured at 570 nm using a microplate reader. IC₅₀ values were determined, and concentrations below IC₅₀ were used for further experiments. Cytotoxicity was assessed at 100, 80, 60, 40, 20, and 1 μg/mL for TiO₂, and 20, 16, 12, 8, 4, and 2 μg/mL for PLGA at 24 and 48 hours. The PLGA + TiO₂ nanocomposite was compared with PLGA alone, TiO₂ alone, and untreated controls.

###  Statistical Analysis

 All the experiments were conducted in triplicate. The results were expressed as mean ± standard deviation (SD). Two-way analysis of variance (ANOVA) followed by post hoc Tukey tests was used to analyze the effects of time, concentration, and formulation type. A *P* value of < 0.05 was considered statistically significant.

## Results

###  FTIR Spectroscopy

**Figure 1:**
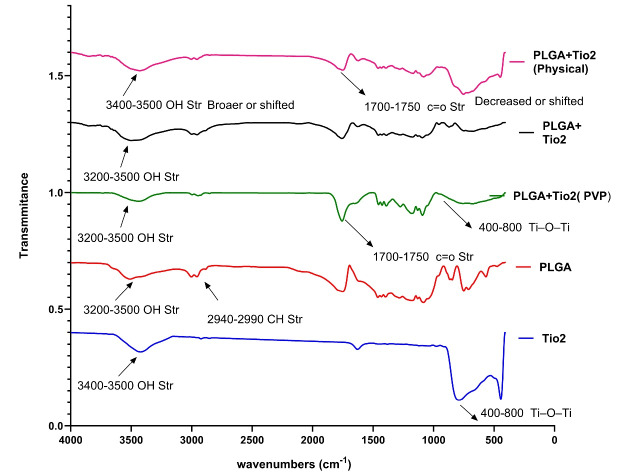
The spectra of FTIR

 FTIR spectroscopy was used to identify functional groups and molecular interactions in the synthesized materials, including pure TiO₂ nanoparticles, PLGA, a physical blend of PLGA and TiO₂, the unstabilized nanocomposite, and the final PVP-stabilized nanocomposite (Figure 1).

 The FTIR spectrum of pure TiO₂ exhibited broad peaks in the region of 400–800 cm ^-1^, which are characteristic of Ti–O–Ti stretching vibrations. A weak, broad signal around 3400 cm-1 was observed, attributable to surface hydroxyl groups, possibly due to adsorbed moisture or surface defects.^19^

 The PLGA spectrum showed a strong absorption band at ~1750 cm ^-1^, corresponding to the C = O stretching of ester groups. Additional peaks were noted at 1180–1080 cm ^-1^ (C–O–C stretching), and 2940–2990 cm ^-1^ (CH₂/CH₃ stretching), which are consistent with its lactide and glycolide components.^20^

 In the physical mixture, all major peaks from both PLGA and TiO₂ were retained without significant shifts in wave numbers, suggesting the absence of chemical interactions and confirming the nature of the sample as a non-reactive blend.

 For the PLGA/TiO₂ nanocomposite without PVP (fast-dried), minor peak broadening was observed in the C = O and Ti–O–Ti regions, which may indicate weak hydrogen bonding or physical entrapment of TiO₂ within the polymer matrix. However, the dispersion appeared incomplete, as indicated by spectral overlap and the presence of sharper peaks.

 The final stabilized nanocomposite containing PVP showed distinct spectral changes. Table 1 presents the FTIR spectral characteristics and the corresponding band assignments. A broader and more intense –OH band was noted at 3200–3400 cm ^-1^, indicating hydrogen bonding interactions among TiO₂, PLGA, and PVP. Additionally, slight shifts in the C = O and Ti–O–Ti bands were observed, indicating successful integration and molecular-level interactions between the constituents. These findings confirm the formation of a chemically interactive nanocomposite in the presence of PVP, while the unstabilized and physically mixed samples exhibited limited or no such interaction. This supports the use of stabilizers like PVP not only for physical dispersion but also for promoting interfacial compatibility between organic and inorganic phases.

###  Particle Size and Zeta Potential with DLS

**Figure 2:**
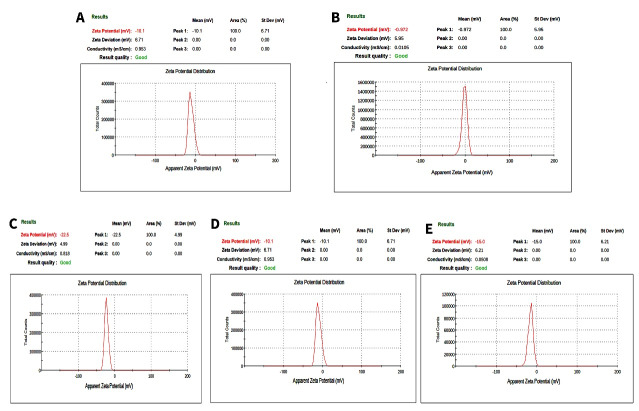
A) Size of TiO_2_ with DLS. B) Zeta potential of TiO_2_. C) Zeta potential of PLGA. D) Zeta potential of PLGA + TiO_2_ without PVP. E) Zeta potential of PLGA + TiO_2_ with PVP

 The average particle size was approximately 15 to 20 nanometers (Figure 2A), indicating nanoscale formation. The monomodal distribution pattern observed in the DLS curve confirmed that the particle sizes were relatively uniform, suggesting a monodisperse system (PDI: 0.21). The measured zeta potential values were recorded as −0.9 mV (close to neutral) for TiO₂, −22.5 mV for PLGA, −10 mV for the PLGA/TiO₂ nanocomposite without PVP, and −15 mV for the PLGA/TiO₂ nanocomposite with PVP (Figures 2B, 2C, 2D, and 2E). The near-zero value observed for TiO₂ indicates that under the applied dispersion conditions (pH and ionic strength of the medium), the particle surfaces carried only a minimal net charge. PLGA exhibited a considerably negative surface charge, consistent with the presence of carboxyl groups along its polymer chains. The reduction in negative charge observed in the nanocomposites can be attributed to the coating or adsorption of TiO₂ onto the polymer matrix, or to the residual stabilizer, which may alter the surface composition of the particles.

###  Morphological Analysis (FESEM)

**Figure 3:**
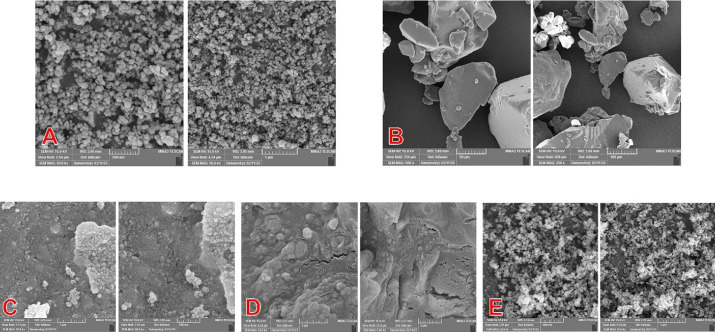
Representative FESEM images: A) TiO_2_. B) PLGA. C) PLGA + TiO_2_ (PVP), D) PLGA + TiO_2_ (without PVP). E) PLGA + TiO_2_ (physical mix)

 FESEM was employed to investigate the surface morphology and particle distribution across different samples, including pure TiO₂ nanoparticles, PLGA microspheres, the physical mixture, the unstabilized nanocomposite, and the final PVP-stabilized nanocomposite (Figure 3).

 The FESEM images of pure TiO₂ revealed uniform, spherical or near-spherical nanoparticles with an average diameter of 20‒30 nm. The particles were mostly discrete but showed minor aggregation, likely due to van der Waals forces during drying.^16^ The smooth surface and homogeneous shape indicated successful synthesis via the sol–gel method. PLGA microparticles appeared as larger, smooth-surfaced, and slightly oval-shaped structures, ranging from 1 to 5 µm in diameter. These particles showed excellent dispersion and no visible clumping, which reflects efficient solvent evaporation during preparation.

 In the physical mixture sample, TiO₂ nanoparticles were seen aggregated on the outer surface of PLGA microspheres. No clear embedding or coating pattern was visible, supporting the interpretation that this formulation lacked true interfacial bonding or structural integration.

 The unstabilized nanocomposite (prepared without PVP and quickly freeze-dried) exhibited a more heterogeneous morphology. TiO₂ particles were irregularly distributed, with some areas of dense clustering. The incomplete encapsulation and non-uniform dispersion may lead to variability in biological behavior and reduced material stability.

 Conversely, the final nanocomposite prepared with PVP exhibited a compact, well-integrated morphology. TiO₂ nanoparticles were uniformly embedded within the PLGA matrix, with minimal surface aggregation. The improved distribution is likely due to the steric stabilization provided by PVP during synthesis, which minimized agglomeration and facilitated better nanoparticle-matrix compatibility.

 These observations are consistent with the FTIR findings and confirm the crucial role of surface stabilization in achieving homogeneous and structurally stable nanocomposites suitable for biomedical applications.

###  XRD Analysis

**Figure 4:**
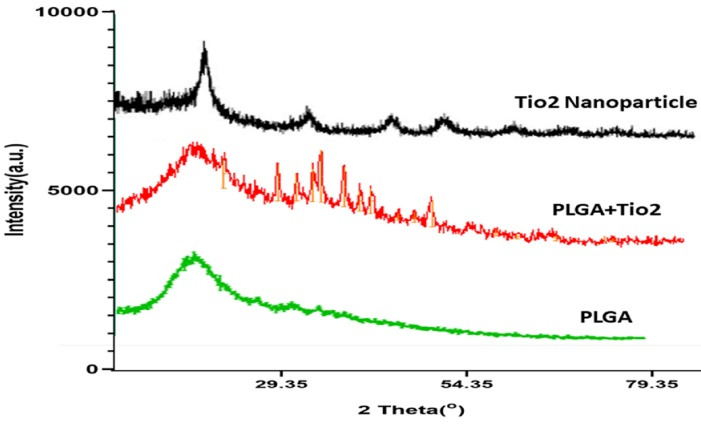
XRD pattern for three samples, including pure PLGA, TiO_2_ nanoparticles, and PLGA/TiO_2_ nanocomposite

Figure 4 presents the XRD patterns of pure PLGA, TiO₂ nanoparticles, and the PLGA/TiO₂ nanocomposite. Pure PLGA exhibited a broad amorphous halo at around 2θ≈18–22°, which is characteristic of its semi-crystalline polymeric structure. In contrast, TiO₂ nanoparticles exhibited sharp, intense diffraction peaks, confirming their crystalline nature and the presence of anatase/rutile phases. The XRD pattern of the PLGA/TiO₂ nanocomposite combined both features: the amorphous background of PLGA and the distinct crystalline peaks of TiO₂. The relative decrease in TiO₂ peak intensity compared to pure TiO₂ suggests uniform dispersion within the polymeric matrix and possible interfacial interactions between PLGA chains and TiO₂ surfaces.

###  TGA Analysis 

**Figure 5:**
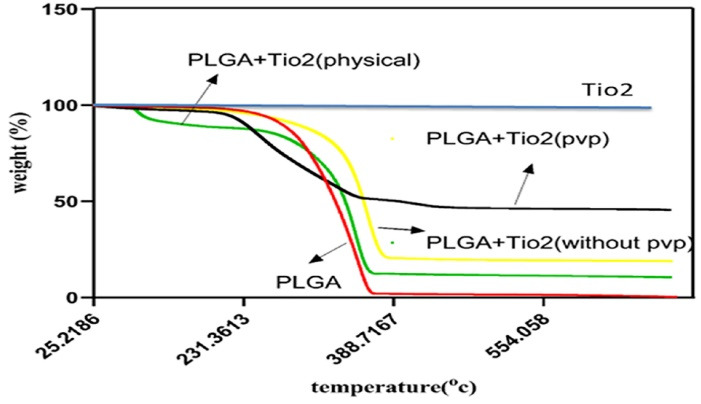
TGA curves

 TGA was performed to evaluate the thermal behavior and stability of pure PLGA, TiO₂ nanoparticles, the physical blend, the unstabilized nanocomposite, and the final PVP-stabilized nanocomposite. As expected (Figure 5), pure TiO₂ showed negligible weight loss across the entire temperature range (25–600 °C), confirming its high thermal stability as an inorganic compound. PLGA, on the other hand, began to degrade at approximately 280°C, with rapid mass loss observed at 300–420 °C, resulting in almost complete decomposition and zero residue at 600 °C. This thermal profile is characteristic of polyester-based biodegradable polymers and reflects the breakdown of ester linkages in its backbone. The physical mixture (PLGA + TiO₂, without any chemical bonding) exhibited a slight improvement in thermal resistance. The onset of degradation was delayed to around 290 °C, and a residue of approximately 25% remained at 600 °C, corresponding to the inorganic TiO₂ content. However, no synergistic effect was observed in terms of extended degradation range or enhanced structural stability.

 The unstabilized nanocomposite (prepared without PVP and dried rapidly) showed a degradation profile similar to the physical mixture, but with a marginally higher residual mass (~30%), suggesting slightly better TiO₂ incorporation. However, its degradation range remained narrow, reflecting limited interaction between the matrix and nanoparticles. The PVP-stabilized PLGA/TiO₂ nanocomposite demonstrated the broadest degradation profile and the highest residual mass (~50%). Degradation began around 260 °C and continued gradually up to 500°C, with delayed peak decomposition and extended stability. This shift in thermal behavior indicates a more intimate interaction between TiO₂ and PLGA, likely mediated by PVP, which enhances nanoparticle dispersion and matrix-nanoparticle bonding.

**Figure 6:**
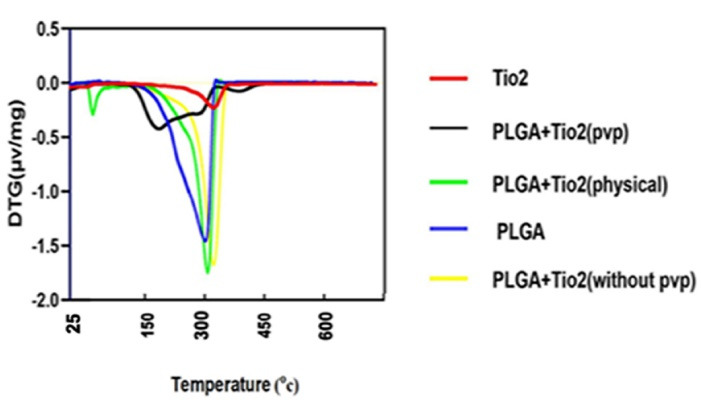
DTG curves

 In addition to the TGA results, differential thermogravimetric (DTG) curves provided further insight into the thermal degradation kinetics of the samples (Figure 6). The DTG peak of pure PLGA appeared sharply around [370 °C], consistent with its rapid thermal decomposition. Upon incorporation of TiO₂, all nanocomposites showed shifts in the DTG peaks, indicating modifications in degradation behavior.

 The physical mixture and the unstabilized nanocomposite exhibited DTG profiles that were relatively similar, with peaks near 380°C, supporting the TGA observation of minimal interaction. However, the PVP-stabilized nanocomposite showed a broadened, slightly shifted DTG peak, reflecting delayed, more gradual degradation, consistent with improved nanoparticle dispersion and stronger matrix-nanoparticle interactions. These DTG findings reinforce the role of PVP in enhancing the thermal stability and structural integration of TiO₂ within the PLGA matrix.

###  In Vitro Cytotoxicity and Biological Evaluation Results (MTT Assay)

**Figure 7:**
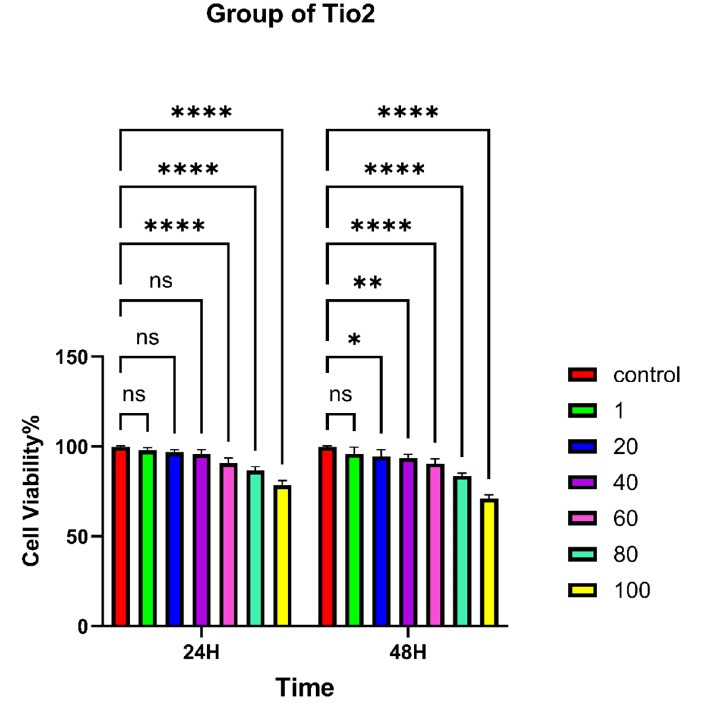
MTT assay results of TiO_2_-treated HGF cells after 24 and 48 hours. Cell viability remained > 60% at all concentrations. No statistically significant differences were observed compared to the control. Statistical significance is denoted as: *P* < 0.05 (*), *P* < 0.01 (**), *P* < 0.001 (***), and *P* < 0.0001 (****)

**Figure 8:**
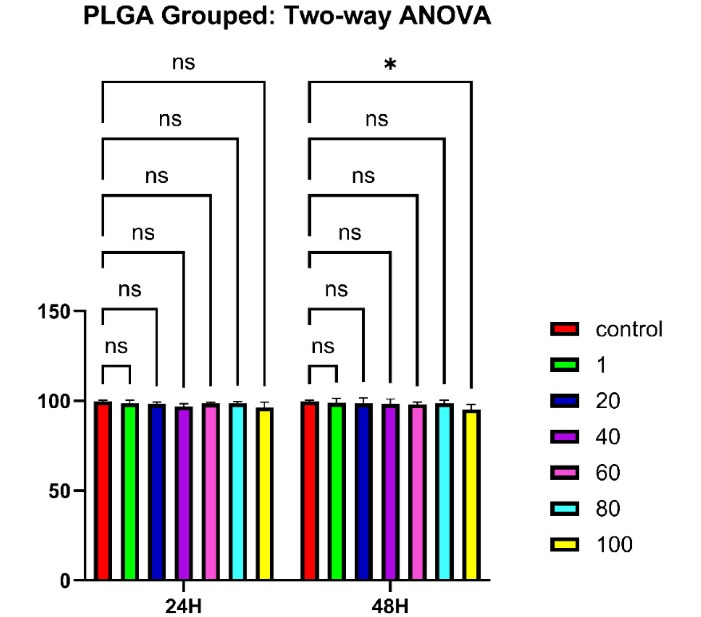
MTT assay results of PLGA-treated HGF cells after 24 and 48 hours. Cell viability remained > 90% at all concentrations. No statistically significant differences were observed compared to the control (*P* < 0.05 [*]), indicating excellent cytocompatibility

**Figure 9:**
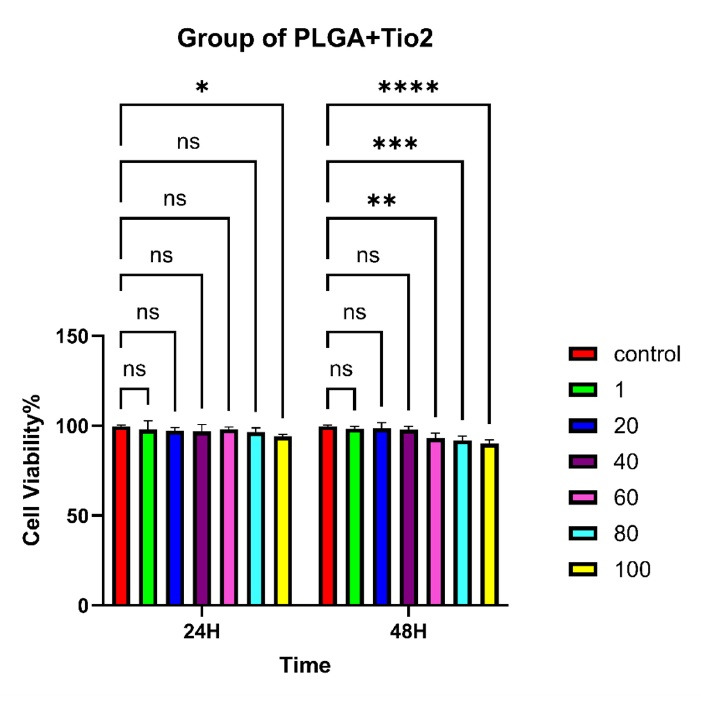
MTT assay results for PLGA/TiO_2_ nanocomposites. A concentration- and time-dependent decrease in cell viability was observed, particularly at 60–100 µg/mL after 48 h. Statistical significance is denoted as: *P* < 0.05 (*), *P* < 0.01 (**), *P* < 0.001 (***), and *P* < 0.0001 (****)

 The MTT assay was used to evaluate the cytotoxicity of different formulations, including pure TiO₂ nanoparticles (Figure 7), PLGA microspheres (Figure 8), and PLGA/TiO₂ nanocomposites (Figure 9), on HGFs. Cell viability was measured after 24 and 48 hours of exposure to various concentrations (1, 20, 40, 60, 80, and 100 μg/mL).

**Table 1:** Characteristic FTIR bands and their assignments

**Sample**	**Wavenumber (cm** - **¹)**	**Assignment/Functional group**
PLGA	~1750	C = O stretching of ester
PLGA	~1180–1250	C–O–C stretching (ester linkage)
TiO₂ nanoparticles	~400–800	Ti–O–Ti lattice vibrations
PLGA/TiO₂ composite	~1750 (shifted)	C = O stretching, interaction with TiO₂
PLGA/TiO₂ composite	~3400 (broad)	O–H stretching, hydrogen bonding
PLGA/TiO₂ + PVP	~1650	C = O and N–C vibrations from PVP
PLGA/TiO₂ + PVP	~3400 (broad)	O–H/N–H stretching, hydrogen bonding (PVP effect)

**Table 2:** Viability of human gingival fibroblasts treated with TiO_2_

**TiO**_2_ ** concentration**	**Time (h)**	**Cell viability (%)** **Mean±S**D
Control	24	99.75 ± 0.53
Control	48	99.75 ± 0.53
1 μg/mL	24	97.99 ± 1.27
1 μg/mL	48	95.99 ± 3.16
20 μg/mL	24	96.85 ± 1.13
20 μg/mL	48	94.60 ± 3.13
40 μg/mL	24	95.84 ± 2.00
40 μg/mL	48	93.59 ± 1.80
60 μg/mL	24	90.73 ± 2.51
60 μg/mL	48	90.56 ± 2.12
80 μg/mL	24	86.64 ± 1.86
80 μg/mL	48	83.73 ± 1.27
100 μg/mL	24	78.47 ± 2.27
100 μg/mL	48	71.09 ± 1.77

**Table 3:** Viability of human gingival fibroblasts treated with PLGA

**PLGA concentration**	**Time (h)**	**Cell viability (%)** **Mean±SD**
Control	24	99.75 ± 0.53
Control	48	99.75 ± 0.53
1 μg/mL	24	98.74 ± 1.48
1 μg/mL	48	98.99 ± 2.15
20 μg/mL	24	98.35 ± 0.95
20 μg/mL	48	98.60 ± 2.71
40 μg/mL	24	97.09 ± 1.21
40 μg/mL	48	98.34 ± 2.29
60 μg/mL	24	98.56 ± 0.51
60 μg/mL	48	98.06 ± 1.16
80 μg/mL	24	98.73 ± 0.84
80 μg/mL	48	98.73 ± 1.52
100 μg/mL	24	96.34 ± 2.58
100 μg/mL	48	96.09 ± 1.07

**Table 4:** Viability of human gingival fibroblasts treated with PLGA-TiO_2_ concentration

**PLGA-TiO**_2_ **concentration (TiO**_2_)	**Time (h)**	**Cell viability (%)** **Mean±SD**
Control	24	99.75 ± 0.53
Control	48	99.75 ± 0.53
1 μg/mL	24	97.99 ± 4.20
1 μg/mL	48	98.49 ± 1.00
20 μg/mL	24	97.35 ± 1.40
20 μg/mL	48	98.60 ± 2.71
40 μg/mL	24	96.84 ± 3.28
40 μg/mL	48	97.84 ± 1.62
60 μg/mL	24	98.06 ± 1.03
60 μg/mL	48	93.06 ± 2.34
80 μg/mL	24	96.48 ± 2.014
80 μg/mL	48	91.98 ± 2.05
100 μg/mL	24	94.09 ± 0.97
100 μg/mL	48	90.09 ± 1.74

 In the TiO₂ group, cell viability decreased progressively with increasing concentration and longer exposure time. Viability dropped below 40% at 80 and 100 μg/mL after 24 hours and decreased further after 48 hours. Notably, after 48 hours, this led to a marked decrease in cell survival. At the highest dose, viability dropped below 70%, indicating significant cytotoxicity (*P* < 0.0001). These results are consistent with previous studies and highlight the potential adverse effects of TiO₂ on mammalian cells at high doses.

 PLGA particles showed excellent cytocompatibility, maintaining cell viability above 90% across all tested concentrations and time intervals. No statistically significant difference was observed compared to the control group. The PLGA/TiO₂ nanocomposite group demonstrated intermediate results. While some reduction in viability was noted at higher concentrations, particularly after 48 hours, the extent of cytotoxicity was significantly lower than that observed with pure TiO₂. After 48 hours, although viability was significantly higher than in the TiO₂ group (*P* < 0.01), it remained modestly reduced compared to untreated controls (*P* < 0.05). This partial cytoprotection confirms the benefits of using biocompatible polymers to improve the safety of metal-based nanomaterials in biomedical applications. Tables 2 and 3 show the viability of human gingival fibroblasts treated with TiO₂ and PLGA, respectively. In addition, Table 4 summarizes the effects of different concentrations of PLGA–TiO₂ on cell viability.

###  Statistical Analysis

 Two-way ANOVA revealed that both concentration and incubation time significantly affected cell viability in the TiO₂ group (*P* < 0.0001), but not in the PLGA group (*P* > 0.05). In the nanocomposite group, while the effects were not statistically significant (*P* > 0.05), the observed trends suggest a potential concentration- and time-dependent response that warrants further investigation.

## Discussion

 The FTIR analysis provided compelling evidence of successful molecular integration between TiO₂ nanoparticles and the PLGA matrix, especially in the presence of PVP. In the stabilized nanocomposite, characteristic bands such as the ester C = O stretching (~1750 cm ^-1^) and Ti–O–Ti vibrations (~400–800 cm ^-1^) remained present, but with slight shifts and broadening, indicating non-covalent interactions—likely hydrogen bonding or dipole–dipole alignment—between TiO₂ and PLGA chains.

 This molecular interaction was not observed in the physical mixture, suggesting that stabilization plays a critical role in nanoparticle distribution and interfacial compatibility. Similar findings were reported by Rasoulian Boroujeni et al, who developed PLGA/TiO₂ porous scaffolds and observed enhanced structural integrity and osteoinductivity due to improved interactions between the inorganic fillers and the polymeric matrices.^21^

 Our findings support the use of stabilizers such as PVP not only to prevent agglomeration but also to facilitate favorable bonding environments at the organic–inorganic interface—key for applications in oral biomaterials, where both stability and biocompatibility are essential.

 The FESEM images showed that incorporating TiO₂ nanoparticles into the PLGA matrix resulted in distinct differences in surface morphology across formulations. The PVP-stabilized nanocomposite exhibited a well-integrated, smooth structure, with TiO₂ particles uniformly embedded and minimal agglomeration. In contrast, the physical mixture and the unstabilized composites exhibited clear signs of nanoparticle clustering, particularly at the surface, indicating poor dispersion.

 These findings are consistent with those reported by Totu et al, who reported that surface modification techniques improved the dispersion of TiO₂ nanoparticles in acrylic resins, resulting in smoother and more homogeneous surfaces suitable for biomedical applications.^22^ Similarly, Eslami et al reported that uniform dispersion of TiO₂ in a PLGA matrix was critical for enhancing mechanical integrity and cellular responses in composite scaffolds used for bone regeneration.^23^

 The importance of achieving homogeneous morphology is paramount in oral biomaterials, where surface roughness and nanoparticle aggregation can affect both bacterial adhesion and host tissue integration. Our stabilized nanocomposite formulation, therefore, holds promise for consistent performance in periodontal and implant-related applications. Although DLS returned a hydrodynamic diameter of ~19 nm, image analysis of FESEM micrographs yielded an average particle diameter of ≈42.9 nm (σ≈40.3 nm, N = 60). The discrepancy can be attributed to differences in measurement principles and sample state: DLS measures particles dispersed in solution and reports a hydrodynamic size (including solvation/adsorbed layers), often with intensity-weighted bias, whereas FESEM measures the projected physical size of dried particles, where partial agglomeration and surface roughness increase the apparent size. Additionally, image segmentation may count small aggregates as single particles, inflating the mean and broadening the size distribution. Overall, both techniques indicate the presence of nanoscale particles; reporting both results and their measurement modes provides a more complete characterization.^24,25^

 Zeta potential results indicate distinct colloidal behaviors among the tested samples. The near-zero zeta potential of TiO₂ suggests a lack of sufficient electrostatic stabilization and a high tendency toward agglomeration under the present dispersion conditions, unless steric stabilization or pH adjustment is applied. In contrast, the negative value observed for PLGA (−22.5 mV) indicates a moderate level of electrostatic stability, consistent with the presence of carboxyl end groups along the polymer chains.

 For the nanocomposite without PVP, the mean zeta potential of −10 mV indicates a reduction in surface charge compared with pure PLGA. This reduction may arise from partial coverage of PLGA by TiO₂ or the adsorption of ionic species during preparation, both of which alter the surface composition. When PVP was introduced, the nanocomposite became more stable, showing a slightly more negative value (−15 mV). Although this is still less electrostatically stable than pure PLGA, the polymeric layer and steric effects provided by PVP are likely to offer sufficient stabilization against aggregation.

 From a biological standpoint, the reduction in negative surface charge may influence protein adsorption and cell–surface interactions, thereby modulating cellular responses, including reduced cytotoxicity. This observation highlights the importance of combining zeta potential analysis with MTT and FESEM results to obtain a comprehensive understanding of the biological performance of these nanocomposites.

 The XRD analysis confirmed that TiO₂ crystallinity was preserved after incorporation into the PLGA matrix, while the polymer exhibited its amorphous halo. The reduction in the relative intensity of TiO₂ peaks in the nanocomposite may be attributed to surface coverage by PLGA and restricted particle aggregation. Similar findings have been reported in previous studies on polymer–metal oxide nanocomposites, where the polymer matrix reduced nanoparticle clustering and enhanced biocompatibility.^26^ These results indicate that PLGA not only provides a biocompatible environment but also mitigates the direct exposure of TiO₂ surfaces, thereby potentially reducing cytotoxicity.

 TGA confirmed that pure PLGA underwent rapid thermal degradation at 300–420 °C, with minimal residue, while the addition of TiO₂ significantly improved thermal stability. The PVP-stabilized PLGA/TiO₂ nanocomposite demonstrated the greatest thermal resistance, with delayed degradation onset and increased residual mass, indicating improved structural stability.^27^

 Eslami et al^23^ reported enhanced thermal properties in PLGA/TiO₂ scaffolds, attributing the improvement to strong interfacial interaction and nanoparticle dispersion. Their findings align with our observations, particularly the correlation between nanoparticle stabilization and heat resistance.

 Although not assessed in the present study, previous work has shown that the inclusion of TiO₂ in polymer-based composites can enhance antibacterial activity and contribute to thermal reinforcement by improving nanoparticle dispersion and interfacial bonding.^21^

 This enhanced thermal performance is especially relevant for dental materials, which must withstand sterilization, processing heat, and variable intraoral temperatures without premature degradation.

 The MTT assay demonstrated that TiO₂ nanoparticles alone exhibited concentration- and time-dependent cytotoxicity against human gingival fibroblasts, consistent with the literature, indicating oxidative stress and membrane disruption at high doses. In contrast, PLGA alone maintained high cell viability ( > 90%), reaffirming its status as a gold-standard biodegradable polymer in oral and craniofacial applications.^28^

 Importantly, the PLGA/TiO₂ nanocomposite exhibited significantly reduced cytotoxicity compared to TiO₂ nanoparticles alone. This protective effect is primarily attributed to PLGA’s role as a biocompatible polymeric barrier, which minimizes direct interaction between the reactive surface of TiO₂ and cellular membranes. The encapsulation of nanoparticles within the PLGA matrix not only improves dispersion but also shields the cells from sharp edges and oxidative stress.

 One key mechanism underlying this reduced toxicity is the suppression of ROS production. Free TiO₂ nanoparticles are known to generate ROS, which can induce oxidative damage, mitochondrial dysfunction, and apoptosis in fibroblasts. However, this suggests that PLGA encapsulation may modulate the surface reactivity of TiO₂ nanoparticles, potentially limiting ROS-mediated cellular injury. However, this mechanism was not directly assessed in this study. In addition, the polymeric coating created steric hindrance, restricting nanoparticle aggregation and uncontrolled cellular uptake—two factors that contribute significantly to nanotoxicity.

 In support of our findings, Xiong et al^29^ investigated the effect of polymeric encapsulation on the cytotoxic behavior of TiO₂ nanoparticles. They reported that coating the nanoparticles with PLGA significantly reduced intracellular ROS levels and mitigated mitochondrial damage in exposed cells. These oxidative mechanisms are among the primary contributors to nanoparticle-induced cytotoxicity, and their suppression by PLGA strongly supports the concept of polymeric shielding as an effective strategy for improving biocompatibility.

 This aligns closely with our observations in the current study, in which the PLGA/TiO₂ nanocomposite maintained high cell viability even at elevated concentrations. It appears that PLGA not only acts as a physical barrier, reducing direct nanoparticle–cell interactions, but also stabilizes the reactive surface of TiO₂, thereby lowering oxidative stress and the potential for inflammation in fibroblasts.

 This mechanism aligns with our previous findings on PLGA–ZnO nanocomposites, in which we observed enhanced cytocompatibility and regulated ion release in human gingival fibroblasts. Mozaffari et al^14^ also reported that polymeric nanocomposites containing metal oxides could support cellular proliferation while mitigating cytotoxicity in vitro.Furthermore, emerging evidence emphasizes that the interfacial chemistry and structural design of nanocomposites play a pivotal role in biological outcomes. The presence of stabilizers, such as PVP, improves nanoparticle distribution and reduces surface charge-mediated interactions, further supporting a favorable biological response. Together, these findings support the use of PLGA-based nanocomposites as safe and effective platforms for oral and periodontal applications.^30^

 From a clinical perspective, our results suggest that the PLGA/TiO₂ nanocomposite, particularly the PVP-stabilized formulation, has excellent potential as a periodontal wound dressing, drug carrier, or implant coating. It combines antibacterial activity (from TiO₂) with soft tissue compatibility (from PLGA), achieving the critical balance needed for long-term success in periodontal therapies.

 Despite the promising results observed in this study, several limitations must be acknowledged. First, all the experiments were conducted in vitro, using a monolayer culture of human gingival fibroblasts. While fibroblasts play a central role in wound healing and are highly relevant for periodontal applications, this model does not fully capture the complex in vivo environment, which includes dynamic interactions with immune cells, extracellular matrix components, and vascular structures. Additionally, only a single cell type was evaluated. The cytocompatibility and biological performance of the nanocomposite may vary when tested against other relevant oral cell lines, such as epithelial cells, osteoblasts, or macrophages. Future studies should therefore aim to incorporate co-culture models and in vivo validation to provide a more comprehensive understanding of the material’s therapeutic potential and clinical safety.

## Conclusion

 This study successfully demonstrated the synthesis and characterization of a PLGA/TiO₂ nanocomposite with favorable physicochemical and biological properties. The structural analyses (FTIR, SEM, and TGA) confirmed the proper interaction between the polymer matrix and TiO₂ nanoparticles, resulting in a thermally stable, morphologically uniform nanocomposite. Cytotoxicity analysis using the MTT assay revealed that pure PLGA was highly biocompatible, whereas TiO₂ alone exhibited dose- and time-dependent toxicity toward human gingival fibroblasts. Importantly, incorporating TiO₂ into the PLGA matrix significantly reduced its cytotoxicity, maintaining cell viability above 80% even at the maximum tested concentration (100 µg/mL). This indicates that PLGA serves as an effective carrier for mitigating nanoparticle-induced cytotoxicity. The findings suggest that the PLGA/TiO₂ nanocomposite holds promising potential for periodontal applications, particularly as a wound dressing or scaffold material where antimicrobial activity must be balanced with cellular compatibility. Future studies should include in vivo testing in periodontal defect models or subcutaneous implantation to evaluate inflammatory responses and regenerative potential.

## Competing Interests

 Currently, Samira Mohammad Mirzapour serves as an Associate Editor for JAPID. The authors declare no other competing interests concerning authorship and/or publication of this article.

## Data Availability

 The data supporting the findings of this study are available from the corresponding author upon reasonable request.

## Ethical Approval

 Ethical approval for this study was obtained from the Faculty of Dentistry, Tabriz University of Medical Sciences (Ethics code: IR.TBZMED.DENTISTRY.REC.1403.408).
